# Magnetohydrodynamic nanofluid radiative thermal behavior by means of Darcy law inside a porous media

**DOI:** 10.1038/s41598-019-49269-9

**Published:** 2019-09-04

**Authors:** Trung Nguyen-Thoi, M. Sheikholeslami, Zahir Shah, Poom Kumam, Ahmad Shafee

**Affiliations:** 1 Division of Computational Mathematics and Engineering, Institute for Computational Science, Ton Duc Thang University, Ho Chi Minh City, Vietnam; 2 Faculty of Civil Engineering, Ton Duc Thang University, Ho Chi Minh City, Vietnam; 3 Department of Mechanical Engineering, Babol Noshirvani University of Technology, Babol, Iran; 4 Renewable energy systems and nanofluid applications in heat transfer Laboratory, Babol Noshirvani University of Technology, Babol, Iran; 5 Center of Excellence in Theoretical and Computational Science (TaCS-CoE), SCL 802 Fixed Point Laboratory, Science Laboratory Building, King Mongkut’s University of Technology Thonburi (KMUTT), 126 Pracha-Uthit Road, Bang Mod, Thrung Khru, Bangkok 10140 Thailand; 6 KMUTTFixed Point Research Laboratory, Room SCL 802 Fixed Point Laboratory, Science Laboratory Building, Department of Mathematics, Faculty of Science, King Mongkut’s University of Technology Thonburi (KMUTT), 126 Pracha-Uthit Road, Bang Mod, Thrung Khru, Bangkok 10140 Thailand; 7 KMUTT-Fixed Point Theory and Applications Research Group, Theoretical and Computational Science Center (TaCS), Science Laboratory Building, Faculty of Science, King Mongkut’s University of Technology Thonburi (KMUTT), 126 Pracha-Uthit Road, Bang Mod, Thrung Khru, Bangkok 10140 Thailand; 8 Department of Medical Research, China Medical University Hospital, China Medical University, Taichung, 40402 Taiwan; 9 Public Authority of Applied Education and Training, College of Technological Studies, Applied Science Department, Shuwaikh, Kuwait

**Keywords:** Mechanical engineering, Nanoparticles, Fluid dynamics

## Abstract

Radiative nanomaterial thermal behavior within a permeable closed zone with elliptic hot source is simulated. Darcy law is selected for simulating permeable media in existence of magnetic forces. Contour plots for various buoyancy, Hartmann numbers and radiation parameter were illustrated. Carrier fluid is Al_2_O_3_-water with different shapes. Outputs prove that conduction mode augments with enhance of *Ha*. *Nu* augments with considering radiation source term.

## Introduction

Transport processes of nanofluid through medium with porosity have been a challenging study in recent times because of its immense applications in geothermal operations, thermal insulations, food processing, and other petrochemical applications. Modeling of nanomaterial flow with imposing Lorentz forces was scrutinized by Yadav *et al*.^1^ and buoyancy force was involved in governing PDEs. A survey present in the literature has shown that thermal properties of nanofluids are better than the usual fluids. Results available have shown that heating properties of solid is larger than liquid. The thermal conductivity engine oil and H_2_ O are thousand times lower than that of copper (Cu). Some preliminary experiments on *Cu*−water suspended nanoparticles are performed by Eastman *et al*.^2^. In the augmentation of heat transmission, Khanafer *et al*.^3^ obtained some interesting results by utilizing nanofluids. The problem studied by Qiang^4^ studied experimentally for copper based water nanofluid and obtained some interesting results. More detail on the investigation of heat transmission with nanofluids can be found in^5–10^. CuO-water based nanofluid inside absorptive medium in the actuality of magnetic force with Brownian motion is performed by Sheikholeslami^11^. MHD fluid flow was portrayed by Raju *et al*.^12^ over a cone. Kolsi *et al*.^13^ employed moved fin to control nanofluid migration through a channel. Different applications of Fe_3_O_4_-water nanofluid were categorized by Sheikholeslami and Rokni^14^. Haq *et al*.^15^ utilized carbon nanotubes with slip flow to improve convective heat transfer.

Nanomaterial flow has received considerable attention from many scientists due to its large uses in engineering^16–18^. Plasma studies and aerodynamics are some practical examples of such flows of radiation mechanism. Radiation is often encountered in frequent engineering problems. Keeping in view its applications Sheikholeslami *et al*.^19–23^ presented the application of nanomaterial in various domains. Some recent publications about heat transfer can be found in^24–32^. To preserve the conduction of about fluid low, nano liquids have been recommended in past ages. Influence electric field on ferrofluid inside a tank with dual adaptable surfaces was demonstrated by Sheikholeslami *et al*.^33^. The investigation of nanofluid with magnetic forces with physical effects and applications can studied from^34–36^. Turbulator effect on swirling nanofluid flow was examined by Sheikholeslami *et al*.^37^. Utilizing such tools make the flow more complex. New model was introduced by Yadav *et al*.^38^ for thermal instability. Furthermore, instability of thermal treatment of nanomaterial within a penetrable zone was exemplified by Yadav *et al*.^39^. They considered variation of nanomaterial viscosity in their simulation. Viscous heating effect on nanomaterial radiative behavior in existence of electric field was scrutinized by Daniel *et al*.^40^. In addition, they considered double stratification with magnetic field. Nanomaterial free convection with double-diffusive was scrutinized by Yadav *et al*.^41^ involving rotation system. Permeable plate with considering radiative impact was modeled by Daniel *et al*.^42^. They imposed Lorentz forces and utilized HAM to solve the problem. Nanomaterial exergy loss with implementation of innovative approach was established by Sheikholeslami^43^. He is expert in this field and shows the approach applications in appearance of magnetic field. Entropy production during transient nanomaterial MHD flow was demonstrated by Daniel *et al*.^44^. They derived governing equations with considering electric field effect. Developments on numerical approach for simulating treatment of nanomaterial were presented in different publications^45–51^.

In current study, effects magnetic force and radiation on migration of nanofluid inside a porous medium was illustrated. CVFEM is considered as tool for showing roles of Rd, Ra, & Ha on performance.

## Problem Explanation

**Figure 1:**
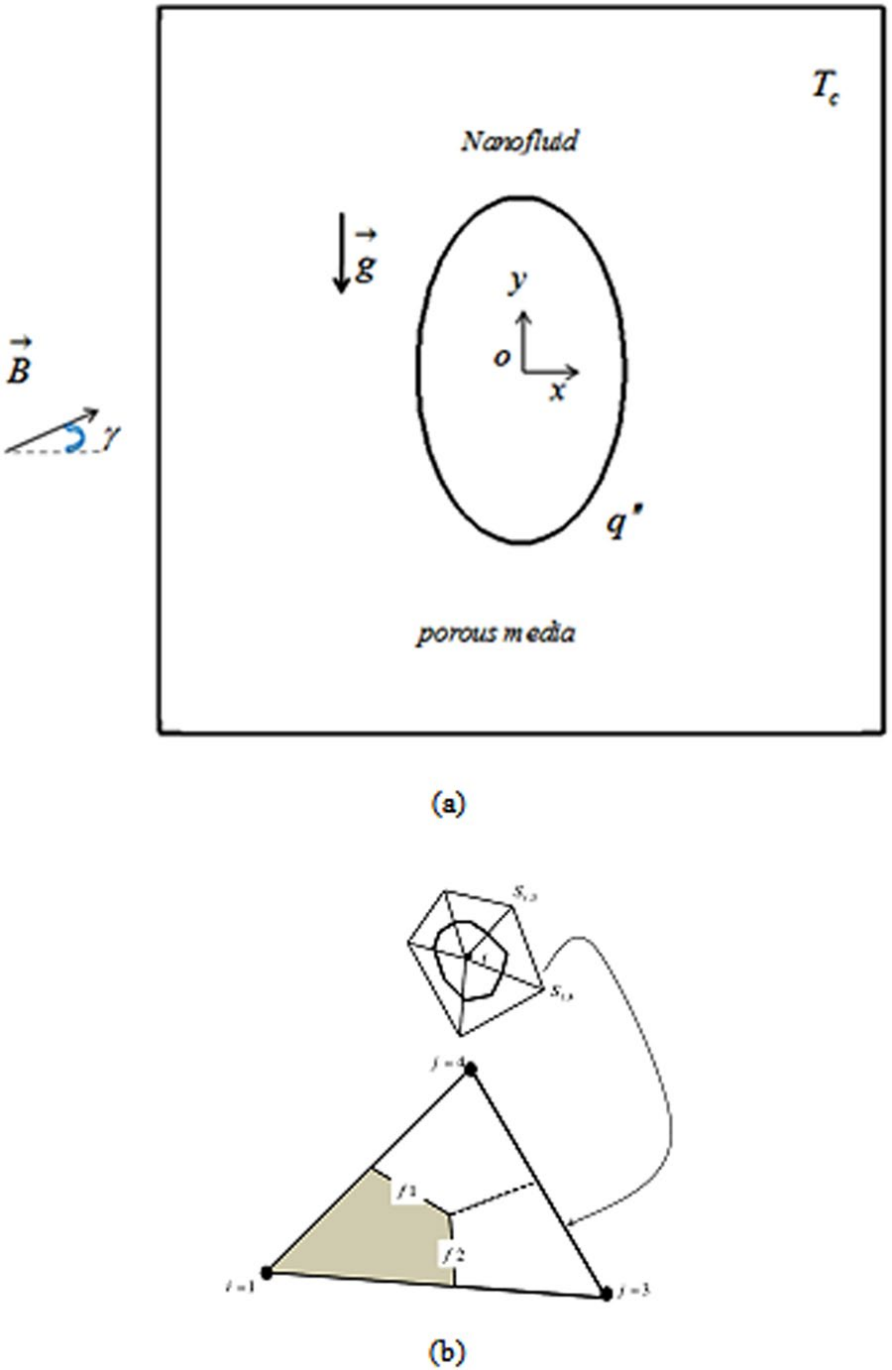
Current porous zone under the impact of magnetic field and sample element.

The shape of enclosure and its boundary conditions have been demonstrated in Fig. 1. Furthermore, example element was demonstrated. Uniform *q*″ was imposed on inner wall. Unchanging magnetic field impact on nanomaterial flow style is surveyed. Porous domain has been full of H_2_O based nanofluid.

### Governing equations and CVFEM

Free convection and radiation impacts on migration of nanofluid inside a penetrable media were pretend under the effect of Lorentz forces. Considering Darcy model, final formulations can be written as:

1 $$\frac{\partial P}{\partial x}=-\frac{{\mu }_{nf}}{K}u+{B}_{0}^{2}{\sigma }_{nf}[\begin{array}{c}-u{(\sin \gamma )}^{2}+\\ (\sin \,\gamma )v(\cos \,\gamma )\end{array}]$$

2 $$\frac{\partial v}{\partial y}+\frac{\partial u}{\partial x}=0$$

3 $$\frac{\partial P}{\partial y}=-\,\frac{{\mu }_{nf}}{K}v+(T-{T}_{c})g{\rho }_{nf}{\beta }_{nf}+{B}_{0}^{2}(\cos \,\gamma )[(\sin \,\gamma )u-v(\cos \,\gamma )]{\sigma }_{nf}$$

4 $$\begin{array}{c}(v\frac{\partial T}{\partial y}+u\frac{\partial T}{\partial x})=-\,\frac{1}{{(\rho {C}_{p})}_{nf}}\frac{\partial {q}_{r}}{\partial y}+(\frac{{\partial }^{2}T}{\partial {x}^{2}}+\frac{{\partial }^{2}T}{\partial {y}^{2}}){(\rho {C}_{p})}_{nf}^{-1}{k}_{nf},\\ \,\,\,\,\,\,\,\,\,\,[{T}^{4}\cong 4{T}_{c}^{3}T-3{T}_{c}^{4},{q}_{r}=-\,\frac{4{\sigma }_{e}}{3{\beta }_{R}}\frac{\partial {T}^{4}}{\partial y}]\end{array}$$

Characteristics of nanofluid have following formulas:

5 $$\begin{array}{rcl}BB & = & \varphi +{(\rho \beta )}_{f}(1-\varphi )/{(\rho \beta )}_{s},BB={(\rho \beta )}_{nf}/{(\rho \beta )}_{s}\\ CC & = & \varphi +(1-\varphi ){(\rho {C}_{p})}_{f}/{(\rho {C}_{p})}_{s},CC={(\rho {C}_{p})}_{nf}/{(\rho {C}_{p})}_{s}\\ {\rho }_{nf} & = & {\rho }_{s}\varphi +{\rho }_{f}(1-\varphi ),\\ \chi -1 & = & \frac{3(A-1)\varphi }{(2+A)+\varphi (1-A)},A={\sigma }_{s}/{\sigma }_{f},\chi =\frac{{\sigma }_{nf}}{{\sigma }_{f}}\end{array}$$

*μ*_*nf*_ & *k*_*nf*_ are represented the Brownian motion forces functions and function of shape factor as mentioned in^52^:

6 $$\begin{array}{rcl}{\mu }_{nf} & = & \frac{{k}_{Brownian}}{{\Pr }_{f}}\times \frac{{\mu }_{f}}{{k}_{f}}+{\mu }_{f}{[1-\varphi ]}^{-2.5},TT=Ln(T)\\ {k}_{Brownian} & = & {10}^{4}\times g^{\prime} ({d}_{p},\varphi ,T)\times 5{\rho }_{f}\varphi \sqrt{\frac{{\kappa }_{b}T}{{\rho }_{p}{d}_{p}}}{c}_{p,f}\\ g^{\prime} ({d}_{p},\varphi ,T) & = & ({a}_{7}Ln({d}_{p})+{a}_{9}Ln({d}_{p})Ln(\varphi )+{a}_{8}Ln(\varphi )+{a}_{10}Ln{({d}_{p})}^{2}+{a}_{6})\\  &  & +\,TT(\begin{array}{l}{a}_{5}Ln{({d}_{p})}^{2}+{a}_{3}Ln(\varphi )\\ +\,{a}_{2}Ln({d}_{p})+{a}_{1}\\ +\,{a}_{4}Ln({d}_{p})Ln(\varphi )\end{array})\end{array}$$

7 $$\begin{array}{rcl}\kappa  & = & ({k}_{f}-{k}_{p}),\\ {A}_{4} & = & \frac{{k}_{f}-m\kappa \varphi +{k}_{p}-\varphi \kappa +m{k}_{f}}{m{k}_{f}+{k}_{p}+\varphi \kappa +{k}_{f}+},\end{array}$$

To get the properties of carrier fluid, we utilized alike model used in^52^. To estimate temperature dependent properties, Rokni *et al*.^53,54^ provide new formulation.

The following non dimensional variables by using of the stream function and, can be gained:

8 $$\begin{array}{rcl}v & = & -\frac{\partial \psi }{\partial x},\Delta T=Lq^{\prime\prime} /{k}_{f},\\ (Y,X) & = & (y{L}^{-1},x{L}^{-1}),\\ \Psi  & = & \psi /{\alpha }_{nf},\,\theta =\frac{T-{T}_{c}}{\Delta T},\\ u & = & \frac{\partial \psi }{\partial y},\end{array}$$

Thus, the last equations are:

9 $$\begin{array}{rcl}\frac{{\partial }^{2}\Psi }{\partial {Y}^{2}}+\frac{{\partial }^{2}\Psi }{\partial {X}^{2}} & = & -Ha\frac{{A}_{6}}{{A}_{5}}[\begin{array}{l}2(\sin \,\gamma ){\Psi }_{XY}(\cos \,\gamma )+\\ ({\cos }^{2}\gamma ){\Psi }_{XX}+{\Psi }_{YY}({\sin }^{2}\gamma )\end{array}]\\  &  & -\frac{{A}_{3}\,{A}_{2}}{{A}_{4}\,{A}_{5}}\frac{\partial \theta }{\partial X}Ra\end{array}$$

10 $$(\frac{{\partial }^{2}\theta }{\partial {X}^{2}})+(1+\frac{4}{3}{(\frac{{k}_{nf}}{{k}_{f}})}^{-1}Rd)\frac{{\partial }^{2}\theta }{\partial {Y}^{2}}=\frac{\partial \theta }{\partial X}\frac{\partial \Psi }{\partial Y}-\frac{\partial \Psi }{\partial X}\frac{\partial \theta }{\partial Y}$$

Important variables can be introduced as:

11 $$\begin{array}{l}Rd=4{\sigma }_{e}{T}_{c}^{3}/({\beta }_{R}{k}_{f}),{A}_{5}=\frac{{\mu }_{nf}}{{\mu }_{f}},\\ Ra=\frac{L{(\rho \beta )}_{f}\,Kg\,\Delta T}{{\alpha }_{f}{\mu }_{f}},\\ {A}_{3}=\frac{{(\rho \beta )}_{nf}}{{(\rho \beta )}_{f}},\,{A}_{2}=\frac{{(\rho {C}_{P})}_{nf}}{{(\rho {C}_{P})}_{f}},\,\,{A}_{6}=\frac{{\sigma }_{nf}}{{\sigma }_{f}},\\ {A}_{1}=\frac{{\rho }_{nf}}{{\rho }_{f}},\,{A}_{4}=\frac{{k}_{nf}}{{k}_{f}},\\ Ha=K\frac{{\sigma }_{f}\,{B}_{0}^{2}}{{\mu }_{f}},\end{array}$$

Inner and outer surfaces have following conditions:

12 $$\begin{array}{ll}\,\,\,{\theta }={\rm{0}} & {\rm{exterior}}\,{\rm{surfaces}}\\ \frac{\partial {\theta }}{\partial n}=1 & \,{\rm{internal}}\,{\rm{surface}}\\ \,\,\,\Psi =0 & {\rm{over}}\,{\rm{inner}}\,{\rm{and}}\,{\rm{outer}}\,{\rm{walls}}\end{array}$$

*Nu*_*ave*_ and *Nu*_*loc*_ have been calculated as:

13 $$N{u}_{ave}=\frac{1}{S}{\int }_{0}^{s}N{u}_{loc}\,ds$$

14 $$N{u}_{loc}=(\frac{{k}_{nf}}{{k}_{f}})\frac{1}{\theta }(1+\frac{4}{3}Rd{(\frac{{k}_{nf}}{{k}_{f}})}^{-1})$$

### Simulation technique, grid and verification

**Figure 2:**
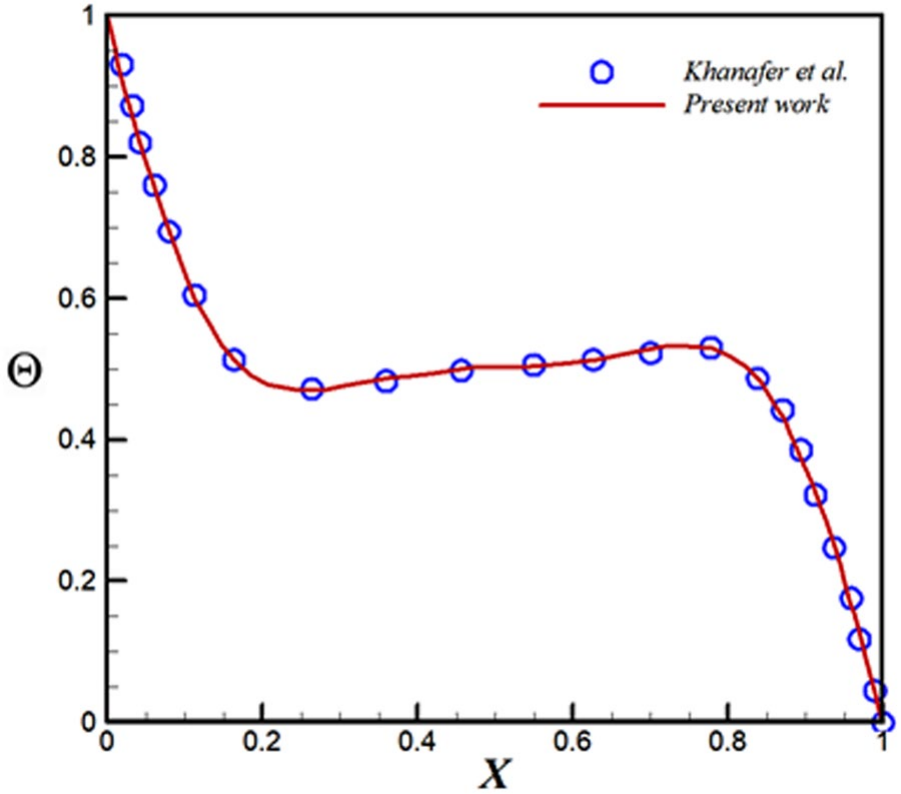
Verification with Khanafer *et al*.^3^ for *ϕ* = 0.1, *Gr* = 10^4^ and $\Pr =6.2(Cu-Water)$.

Combine of two influential approaches has been assembled in CVFEM. As explained in ref.^33^ and shown in Fig. 1(b), such grid is applied in CVFEM. Final equations have attainment to values of *θ*, Ψ by using of Gauss-Seidel technique. Table 1 exhibits the sample for grid management. This procedure should be done because last result should be immaterial of grid size. Verifications of current code for nanofluid convective flow are displayed in Fig. 2 ^3^. These observations show nice accuracy of CVFEM code.

**Table 1:** Mesh study for case of *Ra* = 600, *ϕ* = 0.04 *Rd* = 0.8, *Ha* = 20.

Nu_ave_	Mesh
2.923911	51 × 151
2.927703	61 × 181
2.931546	71 × 211
2.934301	81 × 241
2.938154	91 × 271

## Outcome and Discussion

**Figure 3:**
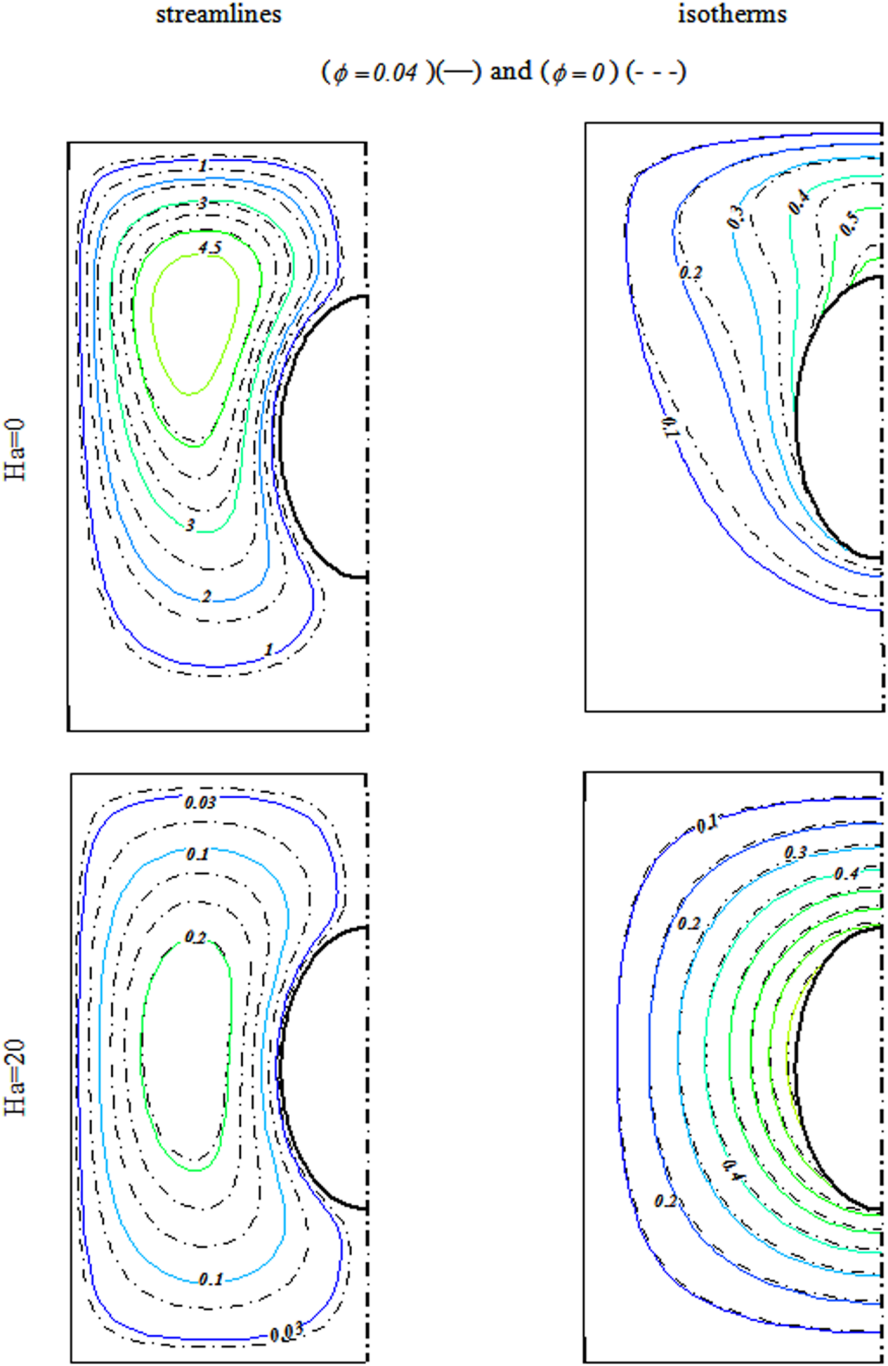
Various of flow style with changing *ϕ* when $Ra=600$, $Rd=0.8$.

**Figure 4:**
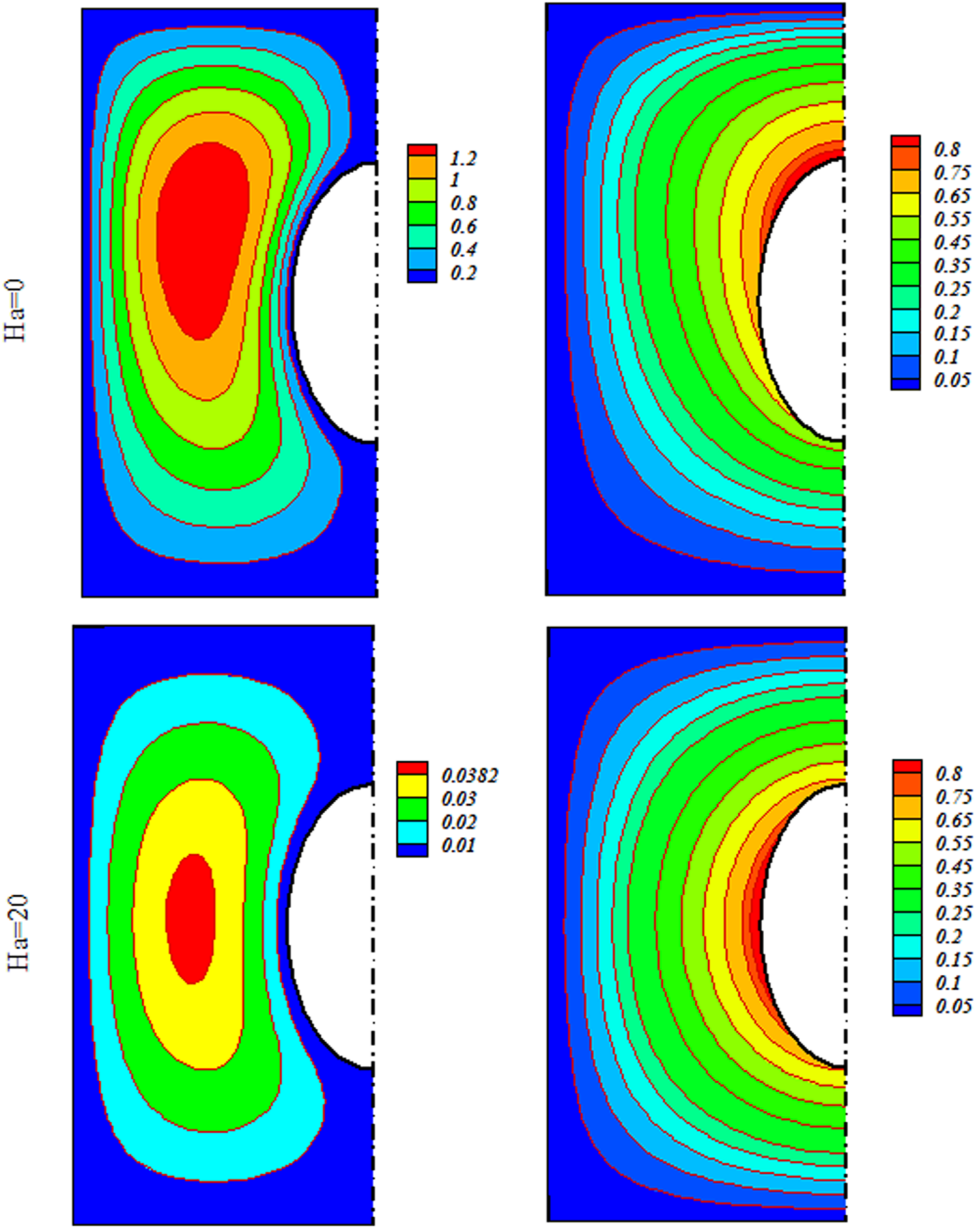
Outputs for various Ha at $Ra=100$, $Rd=0.8$.

**Figure 5:**
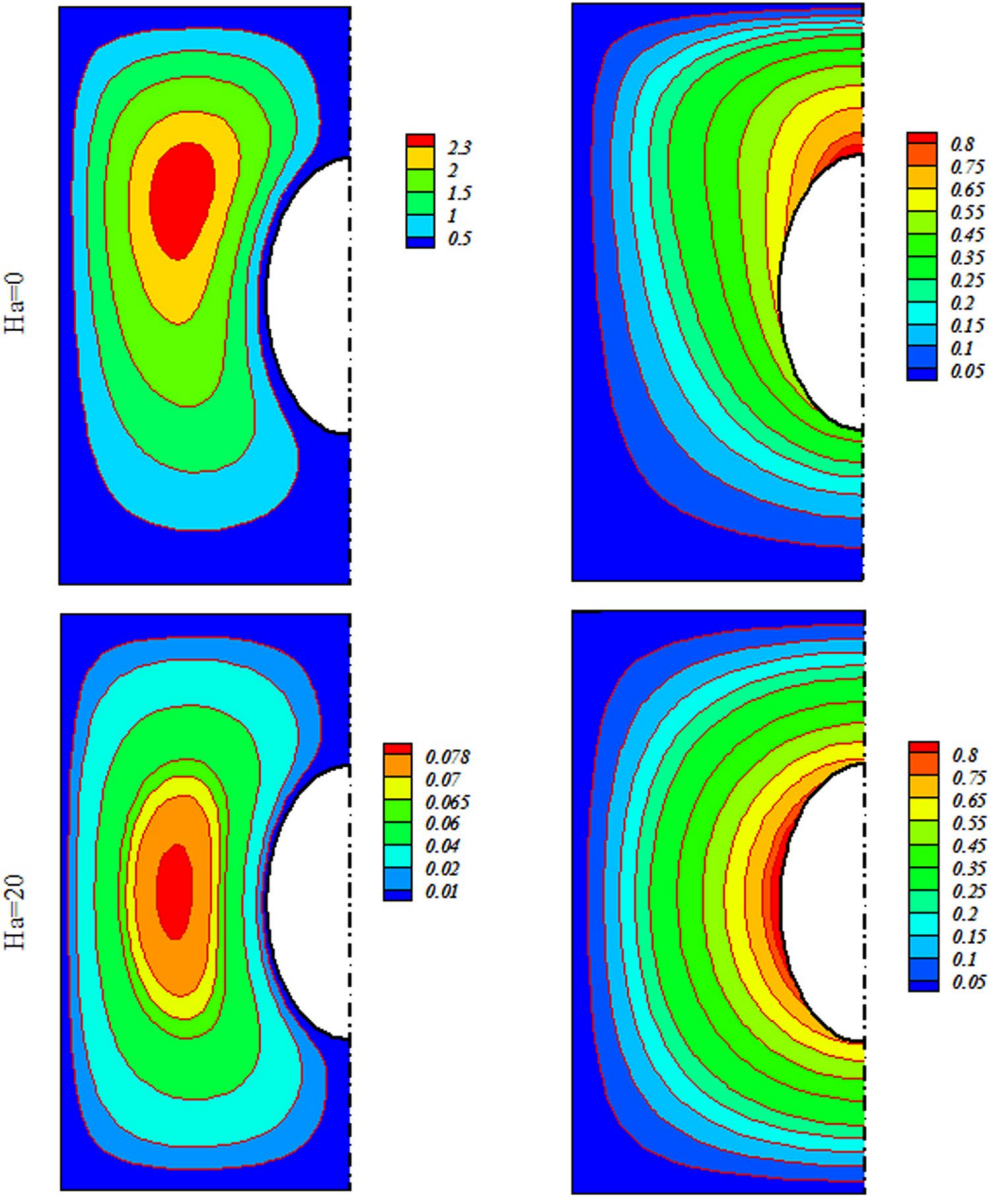
Outputs for various Ha at $Ra=200$, $Rd=0.8$.

**Figure 6:**
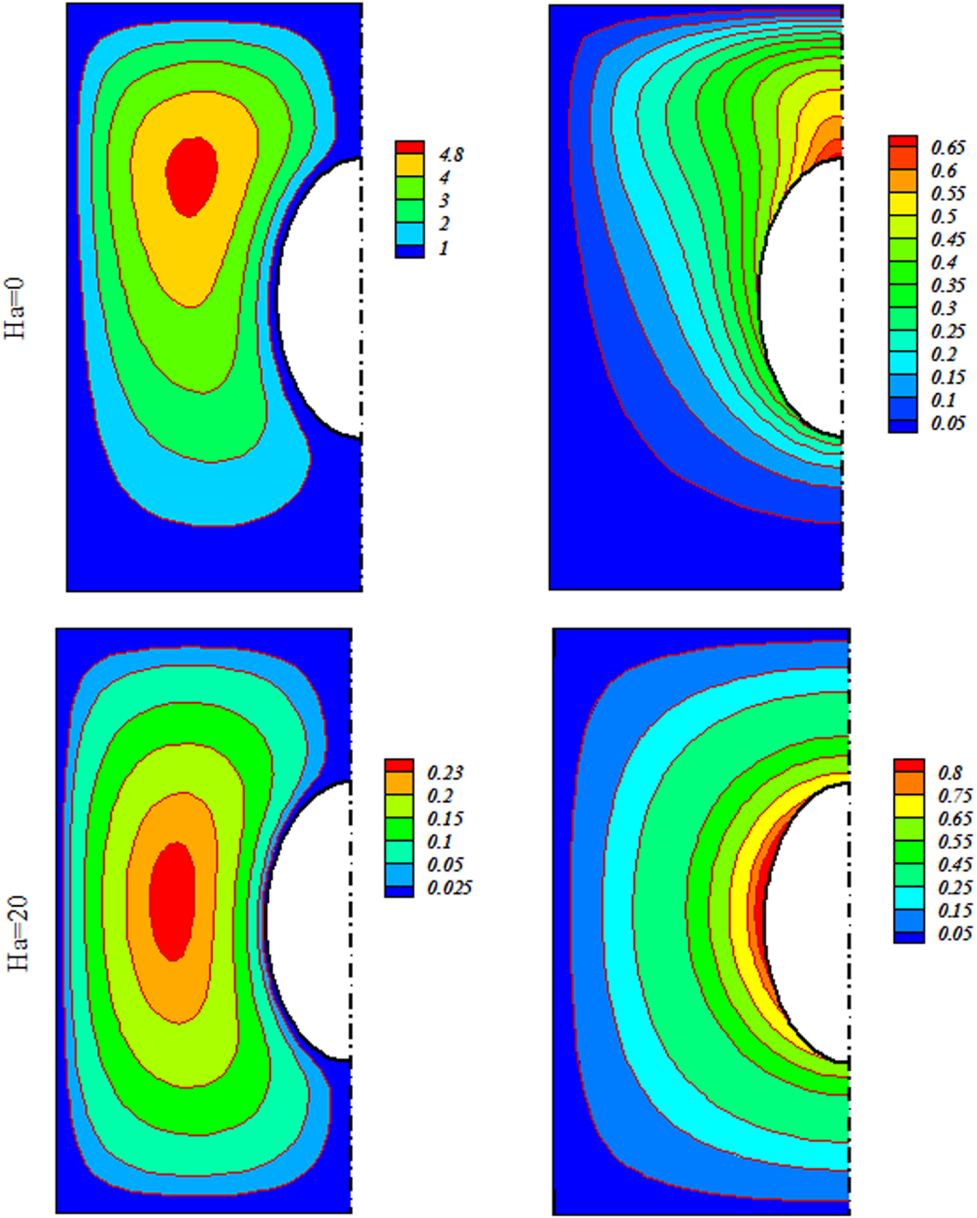
Outputs for various *Ha* at $Ra=600$, $Rd=0.8$.

**Figure 7:**
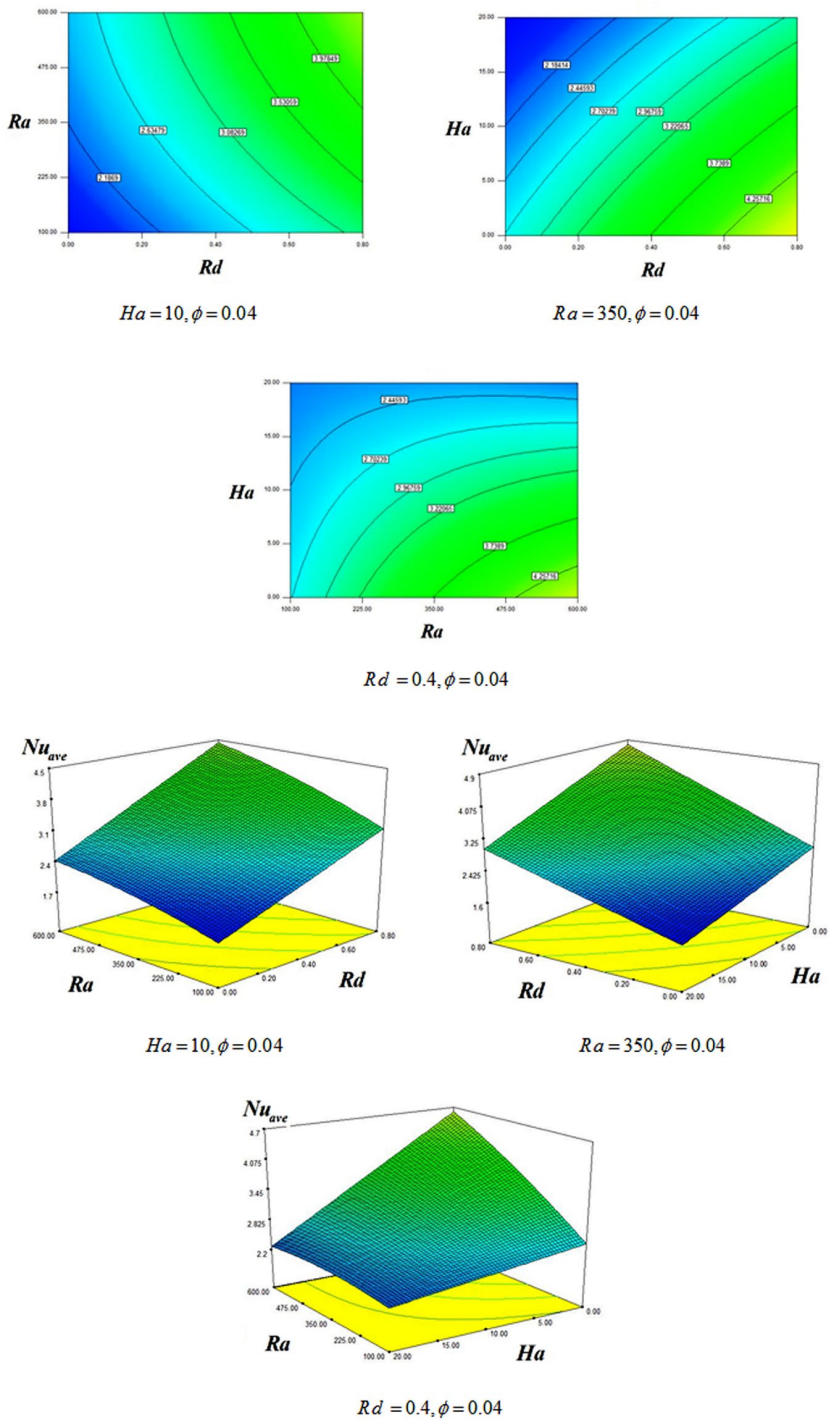
Changes in *Nu*_*ave*_ for various *Rd*, *Ra*, *Ha*.

Radiative nanofluid heat transmission through a penetrable enclosure by means of Darcy law was displayed. Effects of Brownian motion and shape factor on nanomaterial behavior were examined. CVFEM was applied to display the variations of Rayleigh number (*R a* = 100 to 600), radiation (*Rd* = 0 → 0.08), Concentration of Alumina (*ϕ* = 0 to 0.*04*) and magnetic forces (*Ha* = *0* to 20). Deviations of Nu respect to *m* are represented in Table 2. Higher value of Nu is described for Platelet shape. Thus, it is designated for more simulations. Role of scattering Al_2_O_3_ in H_2_O have exemplified in Fig. 3. It is observed that $|{\psi }_{\max }|$ and Nu enhances by diffusing Al_2_O_3_. Since Lorentz force acting, the impact of *ϕ* on isotherms is not important. Impacts of substantial parameters on isotherms and streamlines are displayed in Figs 4, 5 and 6. $|{\psi }_{\max }|$ rises with increase of buoyancy effect while it diminishes with escalation of *Ha*. Simulations for higher Ra leads to complex shape of isotherm with imposing greater buoyancy forces and thermal plume appears. Imposing Lorentz forces make suppress the plume and isotherms force to being parallel to each other’s. For better description, below formula was derived and Fig. 7 was displayed.

15 $$\begin{array}{c}N{u}_{ave}=3.05+0.85Rd+0.49Ra-0.7Ha+0.14Rd\,Ra-0.18Rd\,Ha\\ \,\,\,\,\,\,\,-0.47RaHa-0.1R{a}^{2}\end{array}$$

**Table 2:** Impact of *“m”* on *Nu*_*ave*_ when *Ra* = 600, *ϕ* = 0.04 *Rd* = 0.8.

Shape	Ha	
	20	0
Cylinder	2.887839	5.886044
Platelet	2.931546	5.9168
Spherical	2.800245	5.826297
Brick	2.834335	5.849228

Greater values of radiation parameter and Ra lead to thinner boundary layer which indicates greater *Nu*_*ave*_. Slender thickness of boundary layer was seen with reduce of Hartmann number which proves reduction effect of Hartmann number on *Nu*_*ave*_.

## Conclusions

Imposing Lorenz forces influence on nanomaterial flow by means of Darcy law inside a porous enclosure is reported. Shape factor role was involved to predict nanomaterial properties as well as Brownian motion. CVFEM modeling was done to find the variations of Lorentz and buoyancy forces and radiation parameter on nanofluid thermal characteristic were demonstrated. The concluded points are given as

- Outputs depict that *Nu* improves with improve of buoyancy force but it decrease with augment of *Ha*.
- Higher value of Nu is described for Platelet shape.
- *Nu* augments with considering radiation source term.
- As *Ha* enhances, the velocity of working fluid decreases.
